# Morpho‐metabotyping the oxidative stress response

**DOI:** 10.1038/s41598-021-94585-8

**Published:** 2021-07-29

**Authors:** Mate Rusz, Giorgia Del Favero, Yasin El Abiead, Christopher Gerner, Bernhard K. Keppler, Michael A. Jakupec, Gunda Koellensperger

**Affiliations:** 1 Institute of Analytical Chemistry, University of Vienna, Währinger Str. 38, 1090 Vienna, Austria; 2 Institute of Inorganic Chemistry, University of Vienna, Währinger Str. 42, 1090 Vienna, Austria; 3 Department of Food Chemistry and Toxicology, Faculty of Chemistry, University of Vienna, Währinger Straße 38-40, 1090 Vienna, Austria; 4 Core Facility Multimodal Imaging Faculty of Chemistry, University of Vienna, Währinger Straße 38-40, 1090 Vienna, Austria; 5 Vienna Metabolomics Center (VIME), University of Vienna, Althanstraße 14, 1090 Vienna, Austria; 6 Research Network Chemistry Meets Microbiology, Althanstraße 14, 1090 Vienna, Austria

**Keywords:** Metabolomics, Cellular imaging, Cancer metabolism, Bioanalytical chemistry

## Abstract

Oxidative stress and reactive oxygen species (ROS) are central to many physiological and pathophysiological processes. However, due to multiple technical challenges, it is hard to capture a comprehensive readout of the cell, involving both biochemical and functional status. We addressed this problem by developing a fully parallelized workflow for metabolomics (providing absolute quantities for > 100 metabolites including TCA cycle, pentose phosphate pathway, purine metabolism, glutathione metabolism, cysteine and methionine metabolism, glycolysis and gluconeogenesis) and live cell imaging microscopy. The correlative imaging strategy was applied to study morphological and metabolic adaptation of cancer cells upon short-term hydrogen peroxide (H_2_O_2_) exposure in vitro. The combination provided rich metabolic information at the endpoint of exposure together with imaging of mitochondrial effects. As a response, superoxide concentrations were elevated with a strong mitochondrial localization, and multi-parametric image analysis revealed a shift towards fragmentation. In line with this, metabolism reflected both the impaired mitochondrial function and shifts to support the first-line cellular defense and compensate for energy loss. The presented workflow combining high-end technologies demonstrates the applicability for the study of short-term oxidative stress, but it can be suitable for the in-depth study of various short-term oxidative and other cellular stress-related phenomena.

## Introduction

The central role of reactive oxygen species (ROS) in tuning cell physiology includes essential regulation of cell functional features and proliferation^1–6^. Oxidative stress is also crucial in pathophysiology, as there is a key interplay between ROS, hypoxia and inflammation during tumorigenesis and progression^7–9^. Intriguingly, exploiting redox homeostasis presents a therapeutic opportunity and can also be used for fighting cancer, as cancer cells are close to their antioxidant threshold, while non-cancerous cells can still extend their antioxidant capacities^10–12^. In addition, redox stress is a prerequisite for complex biological phenomena, such as endoplasmic reticulum stress and immunogenic cell death^13^ and for redox activation of anticancer prodrugs^14^. Moreover, uncontrolled ROS production was shown to accompany toxicity as exerted by environmental pollutants^15,16^, food contaminants^17,18^, or therapy^19^. Upon such induction of cellular stress, cell fate is determined by the nature and the strength of the stimulus, as well as typically by the type of cell. Additionally, the coordinated interplay of response pathways and regulatory mechanisms is decisive for resisting and restoring perturbed homeostasis, such as the delicate balance between (oxidative) damage, repair, defense, autophagy and reprogramming. Nevertheless, integrity cannot be maintained, despite adaptations, above a critical threshold and the reprogramming changes from the support of survival to induction of cell cycle arrest, senescence, or some form of cell death (apoptosis, necrosis, autophagic cell death)^20–22^.

Under physiological conditions the bulk of ROS originate from the mitochondria, as side products of aerobic respiration, thus representing a possible source of internal stress. All these aspects make the study of redox stress in a mitochondrial context relevant, both in a functional, spatial and mechanistic perspective. Nevertheless, informative measurement remains challenging.

Metabolomics can capture the molecular signature of the phenotype and thus provides the most functional information^23^. It offers an interrogation window into both cellular energy and redox state. Mass spectrometry-based cellular metabolic phenotyping proved to be powerful in diverse cutting-edge applications ranging from disease etiology to toxicology. While the toolsets are well established for cellular studies using > 100,000 cells, organelle-specific metabolomics is still in its infancy. The challenge lies in the sample preparation step. Typically, organelle fractionation requires conditions jeopardizing unbiased metabolomics. Therefore, most mitochondrial studies lack comprehensiveness so far, e.g., a recent study introduced NADPH imaging at subcellular resolution based on autofluorescence^24^. Using selective redox probes, only specific aspects of redox status (e.g., the redox pair GSH/GSSG) are unraveled, while secondary effects on metabolism and cellular status remain hidden. Recently, mitochondrial metabolomics on advanced cell models obtained by recombinant DNA technology was proposed. A seminal study by Chen et al.^25^ allowed to selectively isolate and extract mitochondrial metabolites. The described method enabled rapid mitochondrial isolation by an epitope-tagged mitochondrial membrane protein.

In this work, we propose an alternative correlative imaging workflow that enables to capture metabolome profiles of mitochondrial ROS response, without mitochondrial isolation steps. More specifically, we combine live cell imaging and metabolomics in a strictly parallel manner. Thereby, the metabolome signatures can be addressed as molecular read-outs of mitochondrial morphological changes. To date, the number of applicable probes in cellular microscopy remains low, despite spectral multiplexing. Thus, integrating state-of-the-art metabolomics can expand the information content of imaging. A proof-of-principle experiment involved cancer cells exposed to hydrogen peroxide, modulating both the redox status and the energy metabolism. The *in-depth* metabolic profile of HCT116 colorectal carcinoma cells upon oxidative stress is obtained by high-resolution mass spectrometry combined with hydrophilic interaction chromatography. We specifically address the accurate determination of redox-sensitive thiol-containing metabolites by derivatization upon extraction. This information was complemented with the morphological characterization of the mitochondrial network obtained with live cell imaging. HCT116 cells are widely used to screen the cytotoxic potency of anticancer agents in vitro^10,26–28^ as well as to investigate the potential effects of bioactive food constituents^29,30^. Here, we aim to pave the way for the application of combined metabolomics and live cell imaging methodology and expand the toolbox to study redox stress in metabolism and cellular morphometric adaptation. In order to demonstrate the applicability of the workflow, we take advantage of the large body of literature in oxidative stress research and seek to capture multiple established notions simultaneously and their possible correlations.

## Results

The correlative metabolomics/imaging method comprised parallel in vitro experiments followed by tailored sample preparation for metabolomics and staining for microscopy. To achieve the highest degree of parallelization, cells were seeded in the same surface density and  ratio of cell number to total medium volume, utilizing live cell imaging compatible multiwell plates and media.

In a proof-of-principle experiment, HCT116 colon cancer cells were exposed to hydrogen peroxide for a short period (2h) in live cell imaging medium and this perturbation could be investigated on both morphological and molecular levels. Microscopic analysis ensured the responsivity of our cell type to the stimulation protocol, albeit maintaining cell integrity and spatial resolution.

**Figure 1:**
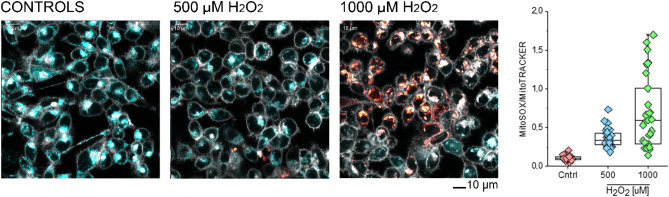
Live cell imaging: effect of hydrogen peroxide treatment. Representative images of the appearance of HCT116 colon cancer cells after staining with MitoSOX (orange, mitochondrial superoxide quantification) and MitoTracker (light blue, mitochondrial morphology). The plasma membrane was counterstained with CellMask (depicted in grey). The increasing MitoSOX to MitoTracker signal ratio upon external hydrogen peroxide treatment (500–1000 µM) in comparison to controls (Cntrl) implies the accumulation of superoxide anions within the mitochondria. Both treatments were different in comparison to controls (Mann–Whitney test; *﻿p* < 0.001).

**Figure 2:**
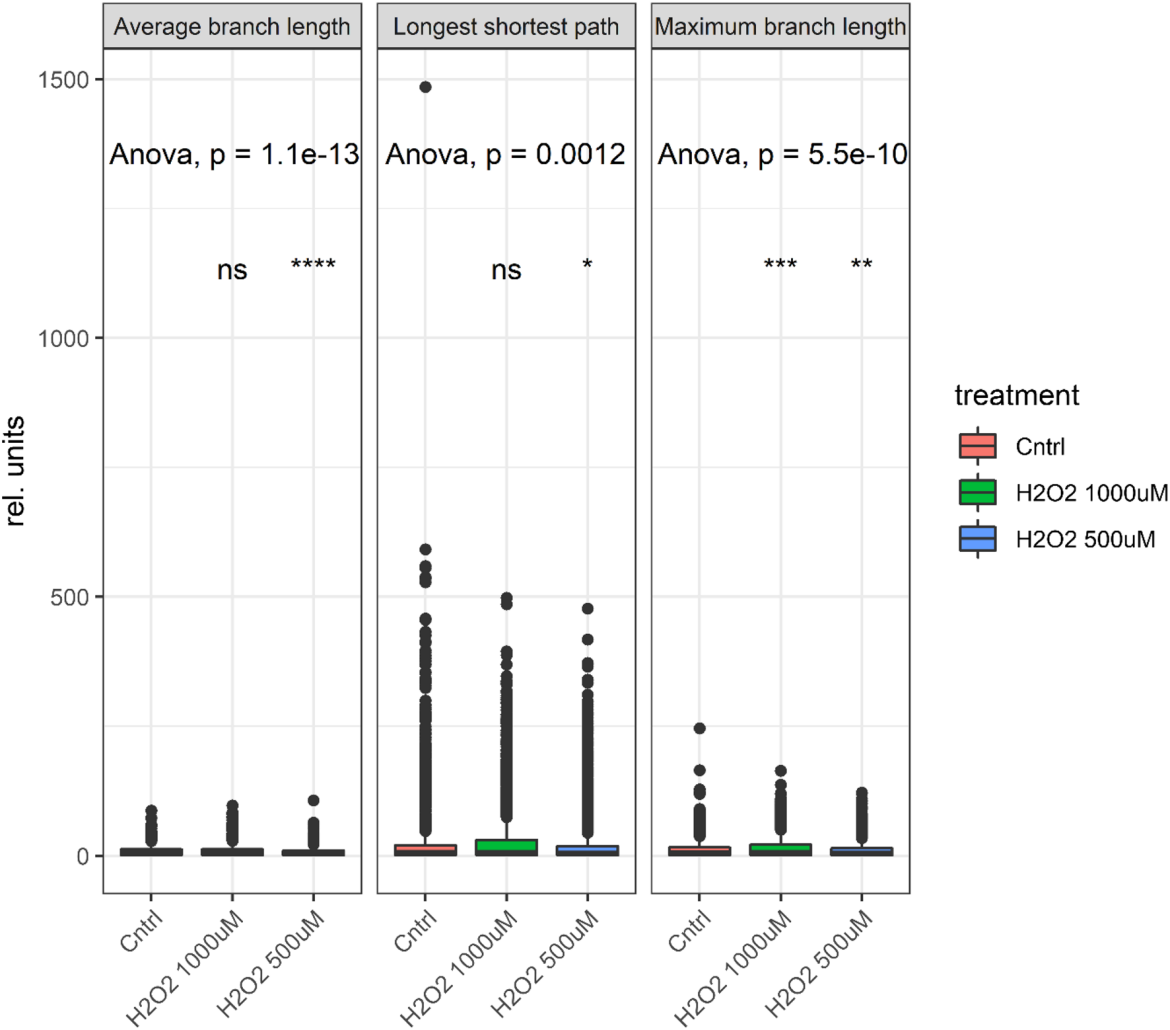
Selected significantly changed morphological features. Average branch length, longest shortest path and maximum branch length in relative units (rel.units) with 1000 µM and 500 µM H_2_O_2_ treatment, and control (Cntrl). Analysis of variance (ANOVA) based on comparison with control. (N = 5790, ns: not significant, *﻿p* > 0.05, *﻿p ≤ 0.05, **p﻿ ≤ 0.01, ***p﻿ ≤ 0.001, ****p﻿ ≤ 0.0001)

A concentration-dependent increase of the mitochondrial superoxide (expressed as MitoSox/MitoTracker signal ratio, Fig. 1) was found. Since structural integrity is tightly related to functional status^31–33^, a multiparametric image analysis of the mitochondrial network was also implemented according to the protocol of Valente and colleagues^34^. Intriguingly, we observed a rather consistent effect of H_2_O_2_ stimulation on the average mitochondrial length and to a certain extent also on the mitochondrial network (ramification and junctions, Fig. 2). These responses seemed more stable at a concentration of 500 µM than at 1000 µM. Indeed, incubation with the highest concentration of H_2_O_2_ was also accompanied by cell morphological changes, possibly accounting for the loss of specificity for some structural parameters at the onset of toxicity and increased mitochondrial density and clustering in the peri-nuclear region^31^. To support this interpretation, a significant decrease in the average and maximal branch length of the mitochondrial network could be measured after incubation with 1000 µM H_2_O_2_.

The parallel metabolomics experiments were performed under an exposure to 500 µM H_2_O_2_ , as a molecular readout for the endpoint of exposure, since the interpretation would be challenged by the observed loss of morphologic specificity at higher concentrations. Sample preparation for metabolomics comprised internal standardization by fully ^13^C-enriched yeast and a derivatization step targeting all thiol-containing metabolites. In our experimental model, metabolome adaptation was substantial despite the relatively short stimulation protocol (2 h). Absolute concentrations were determined for 100 + metabolites covering multiple relevant pathways of the primary metabolome. Among others, energy metabolism, DNA synthesis, TCA cycle, glycolysis, pentose phosphate pathway, amino acid metabolism, and glutathione synthesis were covered in-depth with this method. Absolute concentrations were obtained using fully labelled internal standards in combination with external calibration. As a novelty, the applied HILIC-HRMS covered metabolites as published elsewhere^35^ together with metabolites along the sulfur pathway, which are key factors for the redox balance of a cell. Primary thiols (glutathione, cysteine, homocysteine and glutamyl-cysteine) were assessed as N-ethylmaleimide (NEM) derivatives to prevent oxidation during sample preparation and storage and to prolong stability^36^. Internal standardization and normalization to the protein content resulted in excellent analytical figures of merit and enabled absolute quantification of > 100 metabolites. Moreover, the presence of fully labeled ^13^C biomass monitored unwanted oxidation artifacts during sample preparation^37^. This material, beyond enabling absolute quantification on LC–MS platforms and compensating for losses during sample preparation, was shown to form GSSG with typical isotopologue patterns (^13^C:^12^C 50:50) as a result of oxidation of fully labelled ^13^C GSH and unlabeled GSH. The final derivatization protocol was quantitative, as free thiols remained < LOD in all samples, and it prevented measuring biased GSH/GSSG ratios, as no mixed isotopologues were measured. Consequently, all observed glutathione oxidation could be attributed to the hydrogen peroxide treatment and was not related to abiotic artefacts from the sample preparation and handling. While oxidized glutathione (GSSG) was below LOD (< 10 pmol) in all eight control samples, the hydrogen peroxide treated samples contained approximately 1 nmol GSSG each.

**Figure 3:**
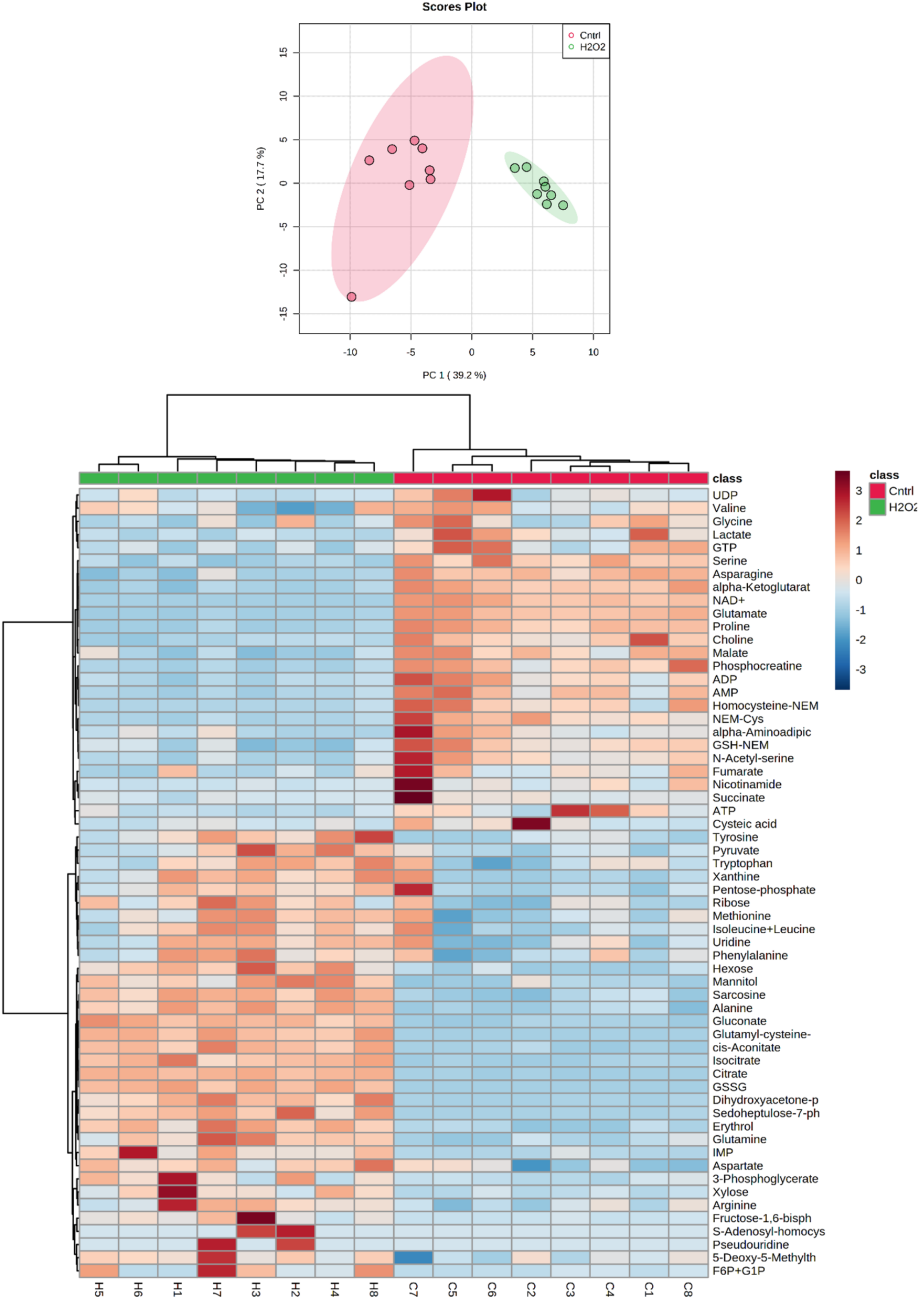
Principal component analysis and heatmap with hierarchical clustering of metabolomics data with 100 absolutely quantified compounds of 500 µM H_2_O_2_-treated samples and controls. The heatmap was constructed with top 60 features based on analysis of variance (ANOVA t-test).

Eight biological replicates were investigated following exposure to 500 µM hydrogen peroxide for 2 h. The data were evaluated in a targeted manner. Exploratory statistical analysis of the absolute amounts of 100 unique compounds using principal component analysis and hierarchical clustering clearly distinguishes within the sample groups (Fig. 3). The first principal component (39.2% explained variance) separates according to treatment, while in the heatmap all the samples are assigned to their respective cluster. Despite the short incubation time, hydrogen peroxide treatment led to strong changes, as a *﻿t*-test with an adjusted *﻿p*-value cutoff below 0.05 resulted in 51 unique significantly altered metabolites (Table 1), from which 30 increased and 21 decreased in their absolute concentrations, including several amino acids, nucleotides, organic acids and carbohydrates or related compounds.

**Table 1:** Significantly altered metabolites upon hydrogen peroxide treatment.

Metabolite	Direction^a^	p-value (adjusted)^b^
**Amino acids and derivatives**		
Glutathione (oxidized)	UP	7.98E−11
Glutamyl-cysteine	UP	9.03E−10
Tyrosine	UP	7.89E−03
Glutamine	UP	4.07E−04
Alanine	UP	3.38E−07
Sarcosine	UP	3.29E−07
Arginine	UP	3.38E−02
Methionine	UP	4.83E−02
Aspartate	UP	1.14E−02
Histidine	UP	3.80E−02
Phenylalanine	UP	2.19E−02
Isoleucine–leucine	UP	1.55E−02
Tryptophan	UP	2.62E−02
Glutathione (reduced)	DOWN	1.06E−03
Asparagine	DOWN	1.58E−06
N-Acetyl-serine	DOWN	3.27E−03
Cysteine	DOWN	1.71E−04
Proline	DOWN	3.33E−08
Glutamate	DOWN	9.03E−10
Alpha-aminoadipic acid	DOWN	4.22E−02
Serine	DOWN	3.54E−06
Phosphocreatine	DOWN	3.79E−05
Homocysteine	DOWN	8.03E−04
**Organic acids**		
Isocitrate	UP	6.73E−08
Citrate	UP	5.74E−14
3-Phosphoglycerate	UP	3.38E−02
cis-Aconitate	UP	2.04E−08
Pyruvate	UP	2.45E−02
Malate	DOWN	1.40E−04
Alpha-ketoglutarate	DOWN	7.11E−08
Lactate	DOWN	6.47E−03
**Carbohydrates and related compounds**		
Sedoheptulose-7-phosphate	UP	4.94E−06
Dihydroxyacetone-phosphate	UP	2.99E−06
Xylose	UP	2.51E−02
Hexose	UP	1.32E−04
Mannitol	UP	1.49E−04
Gluconate	UP	1.55E−09
Erythrol	UP	4.01E−06
Ribose	UP	2.81E−02
**Purines and pyrimidines**		
IMP	UP	3.71E−03
Inosine	UP	2.45E−02
Xanthine	UP	2.02E−02
5-Deoxy-5-methylthioadenosine	UP	2.98E−02
Nicotinamide	DOWN	1.63E−02
AMP	DOWN	1.57E−04
ADP	DOWN	8.90E−04
GTP	DOWN	2.02E−02
ATP	DOWN	3.38E−02
NAD+	DOWN	1.69E−09
**Amines**		
Betaine	DOWN	3.96E−02
Choline	DOWN	1.32E−04

After 2 h incubation in HCT116 adenocarcinoma cell culture (N = 8, N = 8), 51 unique metabolites changed, from which 30 had higher (UP) and 21 lower (DOWN) concentration in means relative to control.

^a^Direction of change in mean relative to control.

^b^False discovery rate adjusted p-value with N = 8 biological replicates.

**Figure 4:**
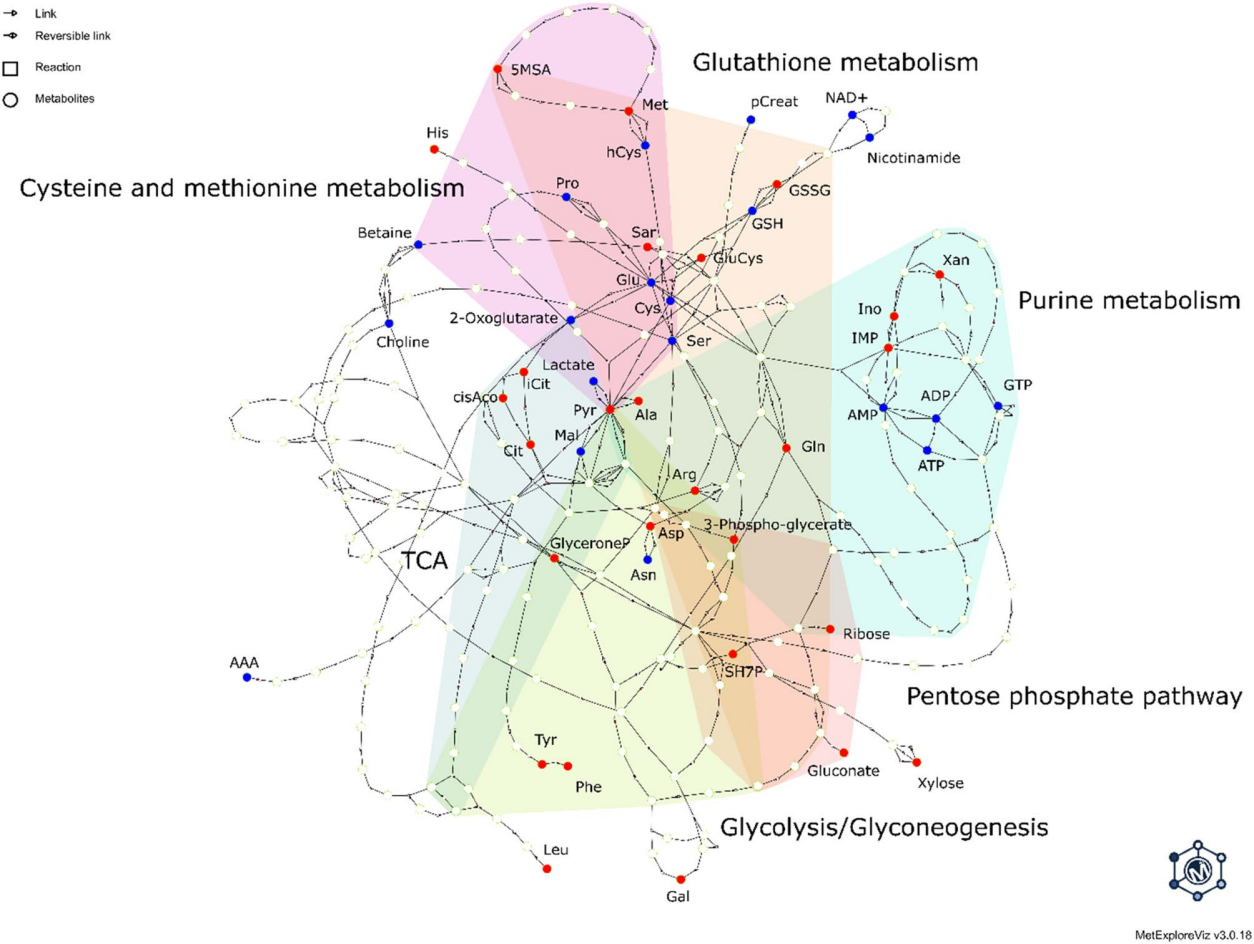
Sub-network constructed from the metabolic network of all possible KEGG reactions, corresponding to the union of all lightest paths between each pair of metabolites in the metabolic fingerprint of redox stress triggered by H_2_O_2_. Metabolites depicted as red nodes correspond to increasing amounts, while as blue nodes to decreasing amounts. Six major distinct pathways where the modulations by redox stress are found are highlighted by different colors. AAA: alpha-Aminoadipic acid, ADP: Adenosine diphosphate, Ala: Alanine, AMP: Adenosine monophosphate, Arg: Arginine, Asp: Aspartate, ATP: Adenosine triphosphate, Cit: Citrate, cisAco: cis-Aconitate, GluCys: gamma-Glutamyl-cysteine, Gln: Glutamine, Glu: Glutamate, GSH: Glutathione (reduced), GSSG: Glutathione (oxidized), His: Histidine, iCit: Isocitrate, IMP: Inosine monophosphate, Leu: Leucine, Mal: Malate, Met: Methionine, 5MSA: 5-Deoxy-5-Methylthioadenosine, NAD+: Nicotinamide adenine dinucleotide, Phe: Phenylalanine, pCreat: Phosphocreatine, Tyr: Tyrosine, SH7P: Sedoheptulose-7-phosphate, Sar: Sarcosine, Ser: Serine, Xan: Xanthine.

In order to further investigate these changes, we constructed a metabolic network with MetExplore^38,39^, mapped the significantly changed metabolites on it and extracted the subnetwork based on the mapping to create a concise, global view of the underlying phenotype upon the short redox stress induced by the hydrogen peroxide treatment. The resulting metabolic network (Fig. 4) displayed strong and consistent changes throughout the citric acid cycle (TCA), glycolysis/gluconeogenesis, glutathione metabolism, cysteine and methionine metabolism, nucleotide synthesis (purine metabolism) and pentose phosphate pathway. Here we show that many of the changes are biochemically close to each other, and only a few possible enzymatic reactions apart. Indeed, the distinct perturbed pathways exhibit a globally coordinated metabolic effort to maintain redox and energy homeostasis and restore cellular damage, as we will discuss below.

## Discussion

Currently, correlative multimodal imaging –omics methods are emerging^40–43^. These include scientific questions for which untargeted analysis can take advantage of high spatial resolution, or when fast kinetic responses are involved, which can be described uniquely by live cell imaging ^44,45^ In this work, correlative imaging was established, integrating mass spectrometry-based metabolomics as an endpoint in live cell imaging experiments on cancer cells. Live cell imaging enables superb spatial resolution and even more importantly, to follow responses under live physiological conditions, which perfectly match the functional readout provided by metabolome analysis. The workflow was applied to study the impact of ROS on metabolism, accompanied by changes in cellular morphology and mitochondrial networks. Oxidative stress, the disturbance of oxidant-antioxidant balance favoring oxidizing environment is largely mediated by a few representative radical molecules. Mitochondria are the main ROS-active cellular sites, mainly due to electron leakage from complexes I or III, resulting in superoxide production, using a large fraction of cellular antioxidant capacity^46^. Under physiological conditions, low levels of ROS are used for cellular signaling and for regulating mitochondrial homeostasis and cellular respiration. We used hallmarks and established notions about oxidative stress response^3,6^ upon hydrogen peroxide exposure with the aim of proving recurring patterns and phenomena in our data generated by the combined imaging –omics method. Perturbation of cancer cells by hydrogen peroxide was selected as a poster case. Although the applied hydrogen peroxide is moderately reactive, it can diffuse into cells or mitochondria through membranes via aquaporins with peroxiporin activity^47^ and generate the highly reactive and toxic hydroxyl radical and superoxide, thereby leading to pathological effects and mutation accumulation.

Pioneering reports on the role of mitochondria in oxidative stress response date back to the last century. Mitochondria are known to handle the extramitochondrial oxidants to a large extent^48,^ thereby tuning the mitochondrial metabolism^49^, in particular glycolysis and oxidative phosphorylation^50^. Furthermore, DNA repair mechanisms^51^ and apoptosis are induced by exogenous^52,53^ and endogenous^54^ hydrogen peroxide and the interplay of calcium fluxes. Oxidative stress and different forms of cellular death^55–57^ have been extensively studied and summarized in exquisite reviews^58,59^. Regarding adaptive metabolism, seminal studies reported on the relevance of NADPH^60,61,^ glutathione^62,63^ and amino acid metabolism^64^. Nrf2 and phase II detoxification were found to have a major role in antioxidant response. Transcriptional regulation^65^ such as NF-kB^66^ and antioxidant enzyme levels^67^ were reported.

In our study, already a short-term exposure to hydrogen peroxide resulted in significantly changed morphology and a perturbed metabolism. More specifically, the external hydrogen peroxide treatment did not uniformly affect all organelles within the cells but primarily affected the mitochondria. Although we introduced H_2_O_2_ to the cell culture medium and only applied a short 2 h incubation, hydrogen peroxide was able to diffuse into the cells and exert its effects, as clearly indicated by both the imaging and metabolomics. A striking result from our investigations is the high and strongly localized MitoSOX to MitoTracker ratio, indicating specific activation of the organelle. We see this despite the exogenous ROS source and the need for diffusion through the cytosol to the mitochondria. A possible explanation for this is that although the exogenous ROS affect the whole cell systematically, the primary effect is the exhaustion of antioxidant capacity, which manifests itself at the mitochondrial site, where the cell is unable to compensate for electron leakage from the ETC. This possible explanation is further supported by the morphological and network analysis of mitochondria. Multi-parametric image analysis revealed a significant reduction of the average and maximal mitochondrial length (Fig. 2), suggesting an imbalance of the fusion/fission equilibrium from mitochondrial network toward single organelles. Mitochondrial fragmentation (fission) represents the first step towards mitophagy and mitochondrial turnover, which is particularly relevant in the stress response to increased oxidative stress^68^. The finding of rapid adaptation to redox stress, as reflected in significant changes at different levels already after short-term exposure, is in accordance with previous studies. Intestinal cells were found to be able to translocate Nrf2 into the nucleus upon oxidative stress or mechanical stimulation within one hour^18^, thus demonstrating that, if required, the oxidative stress response machinery can be activated quite promptly. Similarly, HT-29 colon adenocarcinoma cells can respond to oxidative stress challenge within one hour from the stimulation and are able to up-regulate the pentose phosphate pathway (PPP), as well as to modulate glycolysis and the TCA cycle^69^. Furthermore, human skin cells also activate PPP and recycle glycolytic intermediates to maximize NADPH production even in response to ultra-short exposure to redox stress within seconds^70^.

Also in this work, the structure of mitochondria was in agreement with the metabolic readout, with increased fragmentation of the mitochondrial network (Fig. 2). A significant decrease in GTP concentrations was observed, which aligns well with changes in the mitochondrial network, considering the dependence on local GTP concentrations, as three GTPases facilitate the fusion and division of mitochondrial membranes. Indeed, it was previously described that the fusion-fission equilibrium mirrors metabolic status: the ramified network (fused form) being prevalent during cell respiration and ATP production^71^, and increased clustering was related to a decrease in OXPHOS efficiency^33^. Indeed, several lines of experimental evidence imply mutual regulation between cellular energetic status and mitochondrial fusion/fission equilibrium^68^, making it even more important to monitor metabolic and morphologic adaptation in parallel.

Cancer cells exhibit lower levels of respiration in favor of aerobic glycolysis as well as remarkable metabolic plasticity in comparison with normal tissue. Despite this, it was previously reported, that the investigated colon carcinoma cell line HCT116 generates about 60% of its total ATP via OXPHOS^72,73^. Therefore, there is still a substantial margin for OXPHOS disruption upon mitochondrial impairment.  Correspondingly, the cellular energy charge decreased upon treatment by 60% in our findings. Interestingly, phosphocreatine concentrations were also substantially lowered, which can help to regenerate ATP during buffer fluctuations and maintain cellular energy homeostasis^74^. This seems plausible, as the synthesis of reduced glutathione is energy expensive and requires two ATP-dependent reactions.

Apart from adenosine nucleotides, multiple other purine and pyrimidine nucleotides have altered concentrations. Damage upon ROS exposure can induce DNA repair mechanisms and de novo nucleotide synthesis^75,76^, and redox stress primarily damages the mitochondrial DNA over nuclear DNA^77^.

The metabolic signature of antioxidant defense was accurately measured. It is also important to note that, in case of such a short-term response, cells primarily rely on the basal expression of ROS scavenging enzymes (catalases, superoxide dismutase, glutathione peroxidase and reductase, etc.) and small molecule antioxidants like reduced glutathione, until transcriptional regulation can be adapted^78^. Not only altered GSH/GSSG ratios (reduced glutathione pools due to H_2_O_2_ treatment, and an increase of GSSG), but also multiple changes throughout the glutathione-synthesis pathway indicate a systematic response. Beyond the first-line response of building the GSSG dimer from its reduced monomer and using its reducing power, cells started to replenish the depleted GSH pools. Changes could be observed in several differentially regulated GSH precursors such as cysteine, glutamyl-cysteine, homocysteine, glutamate. These findings were also in perfect agreement with the live cell imaging analysis of the mitochondrial superoxide (Fig. 1). Additionally, the closely related cysteine and methionine metabolism was widely perturbed.

Regarding NADPH/NADP and NAD/NAD ratios, key indicators of the cellular energy state were not considered in this work, as tailored sample preparation is needed for their accurate analysis^79^. However, the indirect effects on pathways were monitored. Upon further redox stress, there is a high NADPH demand for restoration of the reducing capacity of several enzymes in antioxidant defense (co-factor of glutathione reductase, catalase). To prioritize NADPH synthesis, glycolytic flux is diverted into the oxidative branch of the pentose phosphate pathway^75,76^, and glycolytic intermediates from the non-oxidative phase of PPP can be recycled via gluconeogenesis^80–82^. Our results are in agreement with such a coordinated interplay as we see strong, consistent changes throughout these pathways. Increased amounts of glycolytic metabolites could also support the energy needs for glutathione synthesis. The other major NADPH producer apart from PPP is malic enzyme^83,84^, which seems to be also involved, as its reaction partners malate and pyruvate are significantly altered as well.

Diminishing NAD+ concentration can introduce a bottleneck in the conversion of isocitrate to alpha-ketoglutaric acid, thereby stalling upstream metabolites in the TCA cycle and increasing citrate, cis-aconitate and isocitrate concentrations, which is exactly what we observed in the experiment. Furthermore, the results correspond well regarding oxidative inhibition and regulation of TCA enzymes due to high mitochondrial redox stress levels^49,85^. The reduced rate of oxidative phosphorylation (which manifests itself by the increased AMP to ATP ratio) is a response to minimize the endogenous ROS generation, as has been presented and discussed in previous works^70,86,87^. Mitochondria play a pivotal role in cellular fate. Preserving cellular physiology demands tight control of mitochondrial fusion and fission, which are key regulating processes of morphology. There is broad evidence that the morphology and bioenergetic status of mitochondria are linked^88^. The cellular energy status is given by the intracellular nucleotide pool and ATP production. The energy metabolism is in turn connected to the cellular reactive oxygen species (ROS) homeostasis, working on a delicate equilibrium between ROS and the cellular antioxidant system. The advancements in understanding reciprocal, responsive processes of morphological regulation and cellular bioenergetics status emphasized the need for methods allowing to dissect these intertwined processes also with regard to the redox status.

Here we demonstrate that precise metabolomics data relying on internal standardization and absolute quantities combined with live cell imaging are suitable for in-depth investigation of complex biological processes like short-term oxidative stress. As all the molecular signature, spatial information and morphology highlight the implication of mitochondria, we hold that this approach is a viable alternative to workflows with selective mitochondrial extraction. Cellular compartment-specific metabolomics is challenging and so far it only rarely solved problems. Conventional organelle fractionation techniques followed by extraction and LC–MS based measurements are known to introduce significant biases, as lengthy times between isolation and extraction hamper rapid quenching of metabolism. Chen et al.^25^ introduced a superb solution for rapid mitochondrial isolation. Their workflow allowed to obtain metabolic profiles of mitochondria, however, as a drawback it relied on the use of genetically modified in vitro models. While our approach cannot pinpoint the cellular compartment for metabolome perturbations, we mutually validated the functional and quantitative information about the cellular state by the orthogonal information of live cell imaging and accurate absolute metabolite quantities. Overall, we were able to recapitulate established notions previously described in regard to oxidative stress in the metabolism, cellular state and morphology, as well as demonstrate the correlation between these effects. Furthermore, without the need to use an epitope-tag, the methodology can easily be extended to any adherent cell line, avoiding at the same time possible negative implications (e.g. interference with the folding of the target protein and biological activity) introduced by the tag.

## Materials and methods

Methanol and water were LC–MS-grade from Fisher Scientific or Sigma Aldrich; ammonium formate, ammonium bicarbonate eluent additives for LC–MS, hydrogen peroxide (H_2_O_2_) and N-ethylmaleimide (NEM) were purchased from Sigma Aldrich.

### Material and methods metabolomics experiment

#### Cell culture

The human colon carcinoma cell line HCT116 was kindly provided by Brigitte Marian, Institute of Cancer Research, Department of Medicine I, Medical University of Vienna. HCT116 cells were cultured as adherent monolayers in 75 cm^2^ flasks (StarLab), McCoy’s 5a medium (Sigma-Aldrich) was used supplemented with 4 mM l-glutamine and 10% fetal calf serum (FCS) (BioWest) without antibiotics at 37 °C under a humidified atmosphere containing 5% CO_2_. Cell culture media and reagents were obtained from Sigma-Aldrich, and all plastic dishes, plates and flasks were from StarLab unless stated otherwise.

#### Experiment: seeding, treatment and extraction

Conditions in the metabolomics and imaging cultivation plates were matched in regard to seeded cell density and available growth medium. For metabolomics 250 × 10^3^ HCT116 cells per well were seeded (N = 8) in a 12-wellplate format (12-well CytoOne, TC-Treated) in 1 mL of McCoy’s 5a medium (Sigma-Aldrich) supplemented with 10% fetal calf serum (FCS) (BioWest) and 4 mM l-glutamine without antibiotics and incubated at 37 °C (StarLab) under a humidified atmosphere containing 5% CO_2_. At the same time for live cell imaging 70 × 10^3^ cells and 291 µL medium per well were seeded into the imaging wells (Ibidi), in order to match the surface area ratio of the metabolomics well and imaging well (3.5 cm^2^ vs 1 cm^2^). This way we intended to achieve a similar degree of confluence at the day of the experiment.

Two days following seeding, cells were still subconfluent. First, the medium was removed, wells were washed with pre-warmed PBS (37 °C) and 500 µM hydrogen peroxide in live cell imaging solution were added, while for controls live cell imaging solution only (also 37 °C pre-warmed) was added. After incubation for 2 h, imaging and metabolomics wells were treated and prepared for measurement simultaneously..

#### Extraction of metabolites

We applied a combination of cold methanol extraction with NEM-derivatization: the extraction solvent consisted of 80% methanol (Fisher Scientific) and 20% aqueous 10 mM ammonium formate adjusted to pH 7 containing 25 mM N-ethylmaleimide (NEM) in order to form NEM-adducts of primary thiols^89^. The extraction solvent was prepared freshly prior to the experiment. Weighed in from NEM powder, dissolved in 10 mM ammonium formate with pH adjusted to 7.0 The solution was mixed with methanol and cooled down at − 20 °C.

Cells were washed three times with phosphate-buffered saline (PBS) (Sigma-Aldrich) (37 °C) and snap-frozen with liquid nitrogen. 20 μL fully ^13^C labeled (U^13^C) internal standard from ISOtopic solutions e.U. (dissolved in 5 mL 10 mM NH_4_FA at pH 7) was added as well as 180 μL extraction solvent consisting of 20% 25 mM NEM in 10 mM NH_4_FA and 80% methanol). Cells were scraped off in the extraction solution and transferred to Eppendorf tubes as described elsewhere^90^. During extraction, the samples were kept on ice. Subsequently, samples were vortexed and centrifuged (14,000 rcf, 10 min, 4 °C) and from each sample 100 µL of supernatant was transferred into a corresponding MS-vial and 50 µL extract was used to collect a pooled quality control sample. Samples were measured directly without evaporation.

The applied LC-HRMS method was adopted from^35^ as described in^91^. In short, a SeQuant ZIC-pHILIC column (150 × 2.1 mm, 5 µm, polymer, Merck Millipore) with a 15-min long gradient under alkaline conditions with eluents 10 mM ammonium bicarbonate, pH 9.2/10% acetonitrile and 100% acetonitrile were used. The measurement sequence was randomized, blank and pooled quality control samples were injected at regular intervals. The high-resolution mass spectrometer Thermo Scientific™ Q Exactive HF™ quadrupole-high field Orbitrap mass spectrometer was being operated in positive/negative ion-switching mode. External calibration with 133 compounds involving U^13^C internal standardization was carried out.

#### Metabolomics data analysis

Targeted analysis of the data was performed with Skyline 20.2 (MacCoss Lab Software), by extracting the [M−H]^−^ and [M+H]^+^ ions with 5 ppm mass tolerance.

Exploratory data analysis was performed with MetaboAnalyst 5.0^92^. Missing values were imputed by 1/5 of the minimum value of the corresponding variable. For multivariate statistical methods the data were auto-scaled. The metabolic network was constructed with MetExplore^38,39^ with *﻿Homo sapiens* (Strain: Global) (Source: KEGG Map, Version: 24/04/2015) selected as BioSource^93,94^. From the 51 significantly changed metabolites (adjusted *﻿p*-value cut-off 0.05, group variance: equal), 48 metabolites were mapped on KEGG, as mannitol, erythrol and N-acetyl-serine could not be retrieved. Following this, a subnetwork was extracted based on the mapping of significant metabolites.

### Material and methods imaging experiments

#### Evaluation of mitochondrial superoxide production and morphology

In parallel to metabolome analysis, cells were incubated for live cell imaging experiments. Reference measurements of mitochondrial superoxide production and morphometric analysis were performed via imaging workflows to accurately monitor cell status with an independent experimental set-up.

Mitochondrial morphology was visualized with MitoTracker dye and mitochondrial superoxide production with MitoSox dye (both from Thermo Fisher Scientific), as previously described^18,31^. For the imaging, we removed the cell culture medium and incubated the cells with staining solutions (1:1000 dilution in Live Cell Imaging solution, Thermo Fisher Scientific) for 15 min. At the end of the staining, cells were rinsed twice with pre-warmed PBS and immediately imaged in Live Cell Imaging solution.

For microscopy acquisition, we used a confocal Zeiss microscope 710\ELYRA system PS.1 equipped with Plan-Apochromat 63×/1.2 water objective for live cell imaging and an Andor iXon 897 (EMCCD) camera. To this aim, ROI (regions of interest) were randomly selected from the MitoTracker images. During this step, the MitoSox channel was temporarily disabled to avoid selection bias. Multiparametric morphological evaluation of the mitochondrial network was performed according to the method of Valente et al.^34^. Data resulted from the quantification of at least three different optical fields/30 ROIs for every experimental condition.

## Supplementary Information

Supplementary Information 1.

Supplementary Information 2.

Supplementary Information 3.

## Data Availability

Metabolomics data (LC high-resolution mass spectrometry-based metabolomics dataset in rawdata have been deposited to the EMBL-EBI MetaboLights database (Haug et al., 2020) with the identifier MTBLS2672. The complete dataset can be accessed here http://www.ebi.ac.uk/metabolights/MTBLS2672. The processed metabolomics data with absolute concentration, the morphological analysis and constructed subnetwork is provided in the supplementary material. Further information about the datasets generated during and/or analyzed during the study are available on request from the corresponding author G.K. (gunda.koellensperger@univie.ac.at).
