# Supplementary material for: Morpho‐metabotyping the oxidative stress response
Source: Sci Rep. 2021 Jul 29;11:15471. doi: 10.1038/s41598-021-94585-8 (PMC8322264; doi:10.1038/s41598-021-94585-8)
Supplement: Supplementary file 1 — Supplementary Information 1. [file 41598_2021_94585_MOESM1_ESM.docx]

# Additional Information

**Supplementary information** accompanies this paper in form of an .xlsx file, where in several tabs the processed metabolomics dataset “S1 Metabolites in pmol”, the results of the multiparametric image analysis “S2 Morphology analysis”, the input for metabolic network construction with MetExplore with KEGG identifiers and fold-changes in “S3 Metabolic network input” is provided. In a separate .xls file “ExportExcel_5070_KEGG_extractedMetabolicNetwork”, the results of the extracted metabolic network by MetExplore can be found.
